# Effects of ankle–foot braces on medial gastrocnemius morphometrics and gait in children with cerebral palsy

**DOI:** 10.1007/s11832-015-0664-x

**Published:** 2015-06-25

**Authors:** Matthias Hösl, Harald Böhm, Adamantios Arampatzis, Leonhard Döderlein

**Affiliations:** Orthopaedic Hospital for Children, Behandlungszentrum Aschau GmbH, Bernauerstr. 18, 83229 Aschau i. Chiemgau, Germany; Department of Training and Movement Sciences, Humboldt-Universität zu Berlin, Philippstr. 13, Haus 11, 10115 Berlin, Germany

**Keywords:** Cerebral Palsy, Ankle–foot bracing, Ultrasound, Gastrocnemius, Muscle contracture, Equinus

## Abstract

**Purpose:**

In children with cerebral palsy (CP), braces are used to counteract progressive joint and muscle contracture and improve function. We examined the effects of positional ankle–foot braces on contracture of the medial gastrocnemius (MG) and gait in children with CP while referencing to typically developing children.

**Methods:**

Seventeen independently ambulant children with CP and calf muscle contracture (age 10.4 ± 3.0y) and 17 untreated typically developing peers (age 9.5 ± 2.6y) participated. Children with CP were analysed before and 16 ± 4 weeks after ankle–foot bracing. MG muscle belly length and thickness, tendon and fascicle length, as well as their extensibility were captured by 2D ultrasound and 3D motion capturing during passive, manually applied stretches. In addition, 3D gait analysis was conducted.

**Results:**

Prior to bracing, the MG muscle–tendon unit in children with CP was 22 % less extensible. At matched amounts of muscle–tendon unit stretch, the muscle belly and fascicles in CP were 7 % and 14 % shorter while the tendon was 11 % longer. Spastic fascicles displayed 32 % less extensibility than controls. Brace wear increased passive dorsiflexion primarily with the knees flexed. During gait, children walked faster and foot lift in swing improved. MG muscle belly and tendon length showed little change, but fascicles further shortened (−11 %) and muscle thickness (−8 %) decreased.

**Conclusions:**

Use of ankle–foot braces improves function but may lead to a loss of sarcomeres in series, which could explain the shortened fascicles. To potentially induce gastrocnemius muscle growth, braces may also need to extend the knee or complementary training may be necessary to offset the immobilizing effects of braces.

## Introduction

Symptomology of spastic Cerebral Palsy (CP) includes, but is not limited to, muscular weakness, overactivity and contracture [1]. Muscular contractures are thought to some degree to reflect muscle tissue that fails to keep up with bone growth [2]. Plantarflexors are typically seriously affected. Apart from altered neural control, they are intrinsically very stiff [3], resulting in equinus, the most common musculoskeletal impairment in CP [4]. Equinus gait compromises balance and is fatiguing, since it requires more activity of the triceps surae [5]. During childhood of children with CP, the loss in passive dorsiflexion is progressive [6]. Thus, muscle contracture of the triceps surae also seems to deteriorate. On a long-term painful bony foot deformities can result. Temporary immobilization of the stretched calf using casts or braces with or without botulinum toxin injections is a popular treatment [7, 8]. Braces are commonly applied in non-rigid deformities. By holding joints near their end-range, progressive contracture should be counteracted and spastic muscles are assumed to untighten and grow at a rate more equal to that of the bone. Eventually, the gait pattern should also improve. Yet, it is unclear how bracing actually affects the muscle morphometrics in spastic equinus deformity.

### Muscle morphometrics in CP

Ultrasound scans provide a non-invasive means to gain information about a muscle’s architecture. It has already been shown that plantarflexor morphometrics in CP are altered with respect to typically developing peers (TD) [9–14]. In case of the medial gastrocnemius (MG), in one of the spastic leg muscles displaying largest volumetric atrophy [15], there is evidence for reduced muscle belly length (*L*_MB_), cross-sectional area and muscle belly thickness (MT) [9]. However, achilles tendon length (*L*_TEND_) appears to be longer [10] while MG fascicle (bundle of skeletal muscle fibres) length (*L*_FASC_) seems shorter than in TD [11, 12]. Concerning the latter, *L*_FASC_, some inconsistencies have been reported [13, 14]. These discrepancies may be partly explained by difficulties in standardizing the musculoskeletal conditions, e.g., the degree of muscle stretch, during the assessment. On a microscopic level, spastic muscle fibres were also found to contain very long sarcomeres, which was interpreted as an inability to add sarcomeres in series with growth [16, 17].

### Potential response to bracing

Casts and orthotics are currently favourable for contracture management [18]. They should keep the plantarflexor’s muscle–tendon units (MTU) in a stretched position. This is assumed to increase *L*_MB_ over time with a concomittant reduction in pathological equinus posture. Manual stretching of spastic MG can indeed transiently increase its *L*_MB_, *L*_FASC_, as well as *L*_TEND_ [19], but the long-term effectiveness of manual stretch remains doubtful [18]. Cyclic stretches by an external, machine-driven device in combination with active training stimulated the MG fascicles to grow longer and become less stiff [20], positively demonstrating the MG’s adaptive potential. By contrast, most braces induce static, low load stretch over prolonged periods and also immobilise the muscle. Knowledge about the morphometric effects of chronic muscle stretch is primarily derived from healthy animals. When muscle from adult animal is immobilised in a lengthened position, sarcomeres have been shown to be added in series [21]. Muscle fibres in CP may thus grow longer in response to bracing. Yet, the fibres’ cross-section could also atrophy because of the immobilising effect [22]. In juvenile, developing animals, experiments point out that primarily the tendon, and not the muscle fibres, lengthens in response to stretched immobilisation [21, 23, 24]. Stimulated tendon growths could in fact reduce the stretch effects on muscle fibres and eventually induce sarcomere loss [23]. Such a scenario could theoretically decrease the MG muscle belly thickness [MT]. Because of cross-sectional atrophy and the pinnated fibre arrangement, the *L*_MB_ could also decrease. In fact, it has been doubted that stretch-immobilization can promote muscle growth in children with CP [25].

The main aim of this study was therefore to longitudinally re-evaluate MG morphometrics in children with CP after a period of ankle–foot bracing. To define the status quo prior to bracing, *L*_MB_, MT, *L*_FASC_ and fascicle angle (FA), as well as *L*_TEND_, in children with CP was contrasted with TD using ultrasound during passive, manually applied stretches. The total extensibility (~strain) of the muscle, fascicle and tendon was compared as well. We hypothesised that children with CP and equinus have shorter and thinner MG muscle bellies, shorter *L*_FASC_, but longer *L*_TEND_ than TD, and that extensibility of the MTU and its components is reduced. After ankle–foot bracing, we expected that passive dorsiflexion would improve, *L*_MB_, *L*_FASC_ and *L*_TEND_ of the spastic MG would be lengthened and extensibility of the MTU and its components would increase. Our second aim was to compare the functional effects of bracing using 3D gait analysis. We expected dorsiflexion to improve during stance and swing, positively affecting foot positioning at ground contact. Walking speed and step length should thereby be increased.

## Methods

### Participants

To be included, children with CP had to be classified as GMFCS I or II and display non-rigid equinus. Non-rigid equinus was defined as tone on the modified Ashworth Scale (MAS) <4 [26] and a lack of passive range of motion (PRoM) smaller than −10° dorsiflexion (with flexed or extended knees). Further exclusion criteria were a lack of passive PRoM greater than −10° of knee flexion from neutral, crouch gait, leg length discrepancies more than 2 cm, any previous surgery to the leg, botulinum toxin injections within 1 year or bracing within 3 months. We thereby consecutively included 17 (9/8 male and female; 9/8 unilaterally and bilaterally involved, 7/10 GMFCS I and II) children with CP (age range 5y 11mo–15y 6mo) from our outpatient department. As a reference group, 17 TD were included (6/11 male and female, age range 6y 0mo–15y 4mo). Only the (more) involved side was analysed in children with CP based on passive dorsiflexion. For TD, one leg was randomly chosen. Institutional ethics approval was granted and all subjects and their parents gave informed written consent.

### Bracing

**Fig. 1:**
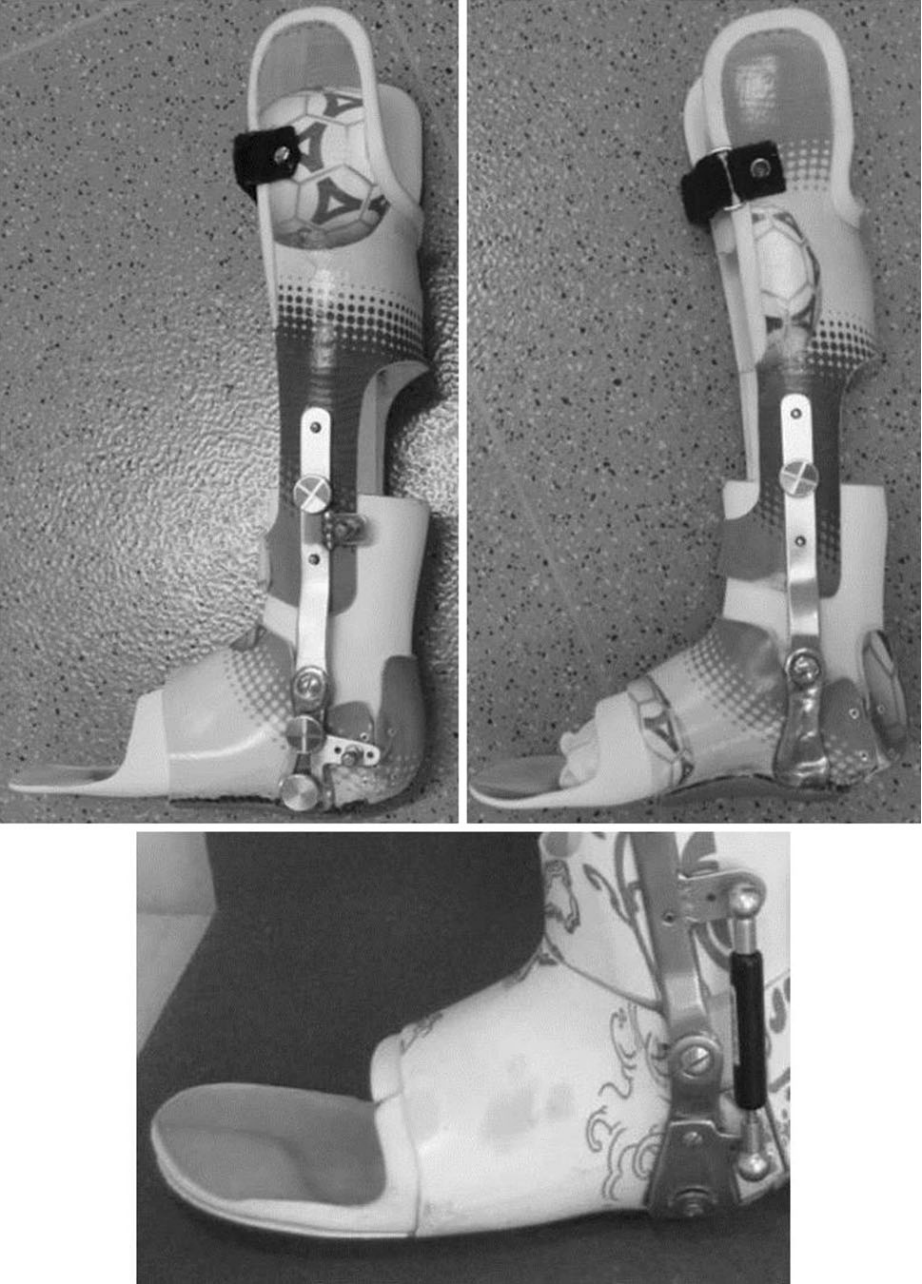
Medial and lateral view of the ankle–foot brace with removable heel cap fixation, subtalar circular locking mechanism and optional posterior gas-spring for further dorsiflexion push-off

An articulated ankle–foot orthotic brace was individually manufactured out of glass and carbon-fibre–reinforced plastics (Fig. 1). The lower leg shell is an S-type calf-construction with condylar support. It is fixed below the tibial tuberosity with a Velcro strap. The foot shell is a circular foot support. Both parts are linked by a constraint metal ankle hinge aligned in maximum passive dorsiflexion, while keeping the knee extended without perceiving intolerable discomfort. The subtalar joint was locked by a circular frame, the heel was fixed with a removable heel cap.

Plantarflexion movement was blocked, and the dorsiflexion RoM was 5–10°. The brace was reviewed every 4–6 weeks and the metal ankle hinge was realigned if possible. If plantigrade position could be achieved and if tolerated, gas springs (~2–3 Nm) were integrated to provide a constant dorsiflexion push during night-wear (seven of 17 children). This resistance could be voluntary attenuated upon mild plantarflexor contraction and all 17 children were intended to wear this brace during sleep. If passive dorsiflexion was less than −5° from plantigrade, they were additionally prescribed daytime use to extend the duration of brace wear. Twelve of 17 children met the criteria for day-time use. Three of those were not compliant with day wear, so that a total of nine (of 17) wore the brace during both day and night. Eight of 17 wore the brace only at night and foot orthotics intended to prevent foot deformities due to mid-foot or subtalar instability during the day.

### Set-up and data collection

All children with CP were analysed before and after bracing. Measurements were performed in the movement laboratory on the day of their outpatient appointments. TD were analysed on a single occasion. Apart from ultrasound scans, all participants were clinically manually examined by the same evaluator and underwent an instrumented 3D gait analysis (3DGA). PRoM for knee extension, popliteal angle (opposite hip flexed) and dorsiflexion with the knee flexed were measured using ruler-based goniometry. Plantarflexor tone was graded on modified Ashworth Scale (MAS) [26]. Passive dorsiflexion with the knee extended was instrumentally measured using motion capture data during MG ultrasound scans.

For 3DGA, a Vicon Nexus system (Vicon, Oxford, UK) with 8 MX-Cameras was used to capture barefoot gait at self-selected speed along a 12 m walkway. Markers were placed according to a modified Plug-In gait Model [27]. Marker data were sampled at 200 Hz and force plate at 1000 Hz via two force plates (AMTI, Watertown, USA). Gait analysis was repeated until five clean strikes on the force plates from each foot could be obtained.

**Fig. 2:**
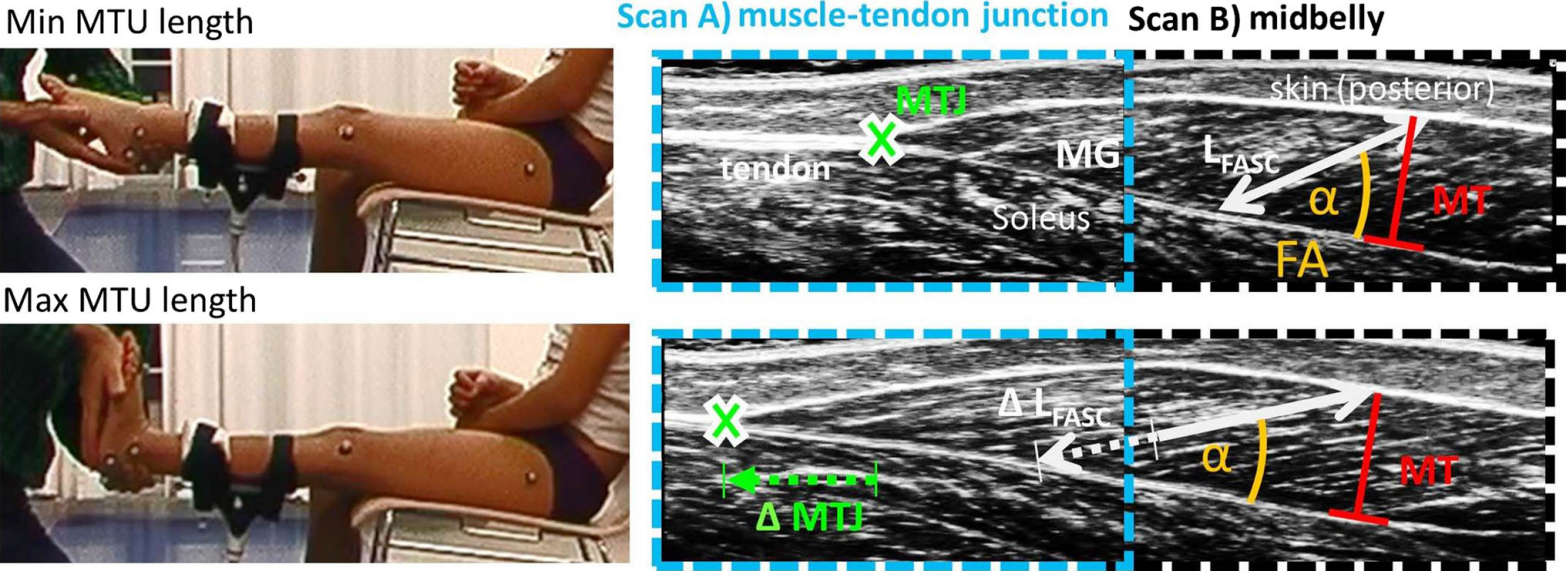
Experimental setup: *Left side* Child positioning with custom-made carbon cast for probe fixation and markers of the motion capture system. *Right side* Superimposed ultrasound scans of the medial gastrocnemius **a** muscle–tendon junction (MTJ), and **b** its midbelly portion with representation of morphometric assessment. *L*_FASC_ fascicle length, *FA* fascicle angle, *MT* muscle thickness

For the ultrasound scans, children were comfortably seated (hip semi-flexed) in a chair (Fig. 2). Retro-reflective markers of the 3D motion capture system remained on the leg (Fig. 2) to track knee alignment and ankle motion during the scan. A 7.5 MHz, 8 cm in width, linear array probe (Sonoline Adara; Siemens, Munich, Germany) was attached with a carbon cast that was equipped with a cluster of four markers. The probe was attached at two locations: over the muscle–tendon junction (MTJ) and over the mid-belly (halfway between popliteal crease and MTJ). The image plane was aligned with the fascicles according to a standardised protocol [28]. The leg was passively lifted and the knee extended as feasible. The same examiner manually moved the ankle slowly and continuously from flexion to extension and back. Prior to data collection, the ankle was preconditioned with three cycles. Then, three to five dorsiflexion stretches were captured while the children could view the ultrasound screen and were encouraged to relax. If muscle contraction was manually sensed as sudden resistance, or whenever contraction was visually apparent, trials were repeated.

### Data analysis

To compare spatio-temporal gait, velocity and step length were extracted and normalised as described by Hof [29], to account for growth. Peak values for ankle dorsiflexion during stance and swing and knee extension during stance were also analysed. The foot landing pattern was characterised by the foot-to-floor angle at ground contact. To quantify ankle kinetics, the peak moments during the first and second half of a stance were selected, as well as the peak power during push-off.

For the ultrasound scans, the position of the markers and the ultrasound movies were continuously captured using Vicon Nexus software with a sampling rate of 200 and 25 Hz, respectively. Subsequent analysis was done in MatLab software (MathWorks, Natick, USA). The MTJ in the ultrasound movies was manually located framewise (Fig. 2). Concerning the fascicles, three to five different mid-belly fascicles were separately localised (straight line between upper and deeper aponeurosis along hyperechoic [bright] collagenous tissue) and an automated tracking algorithm was used to continuously track their elongation during stretch [30]. MT was measured at minimum and maximum stretch only. MT was defined as the distance between the upper and deeper aponeurosis, perpendicular to the deep aponeurosis [10], located halfway between popliteal crease and MTJ. FA was calculated as: α = arcsin(MT/*L*_FASC_). The distal *L*_TEND_ was defined as a straight-line from the heel marker to the MTJ. Since the entire Gastrocnemius MTU could not be tracked directly, *L*_MTU_ was calculated using previously established equations relying on motion capture data concerning tibia length, knee and ankle angles, as well as on individual anthropometrics [31]. *L*_MB_ was calculated as *L*_MB_ = *L*_MTU_–*L*_TEND_ [10]. *L*_FASC_ was represented by the average of all fascicles. For each trial, MTU stretches (from minimum to maximum length) were separated. To represent the average *L*_FASC_, *L*_MB_ and *L*_TEND_ lengthening across the *L*_MTU_ stretch for each individual, data of each stretch was split into ten equally spaced steps. Finally, the averages at these query points were taken before a third-order polynomial was fitted. For *L*_MTU_, *L*_MB_, *L*_FASC_ and *L*_TEND_, minimum and maximum values were extracted. Besides, *L*_MB_, *L*_FASC_ and *L*_TEND_ were analysed at similar degrees of MTU stretch. Since there was no common overlap in *L*_MTU_ between all participants, the midrange *L*_MTU_ (halfway between minimum and maximum stretch) was first calculated for each individual with CP before bracing. To standardise comparisons, the average midrange *L*_MTU_ from children with CP was used for TD. To compare morphometrics before and after bracing, midrange *L*_MTU_ could be exactly matched individually. All parameters were normalised to shank length, defined as from the malleolus to the knee marker. Their extensibility was calculated as  % change between minimum and maximum length.

### Statistics

Shapiro–Wilk tests were used to test normality. At baseline, children with CP were compared with TD. Statistical group differences were evaluated with independent *t* tests. To compare children with CP before and after bracing, paired *t* tests were performed. Mean differences and 95 % confidence intervals were calculated. Alpha-level was set at 0.05 (two–sided tests). Standardised effect sizes were expressed as Cohen’s *d*. Threshold values were 0.2, 0.5 and 0.8 for small, medium and large effects. Unless indicated differently, values are presented as mean (±1 SD).

## Results

### Participant characteristics and clinical exam

Values for TD and CP and the test statistics are summarised in Table 1. There were no significant differences in age, height, shank length or mass (*p* > 0.279). Children with CP demonstrated significantly shorter popliteal angles (*p* < 0.001). Average passive dorsiflexion in CP with knees flexed [8° (11°)] and extended [2° (10°)] was considerably reduced with respect to [TD 29° (8°)] and [15° (5°)], all *p* < 0.01. At Follow-up, on average 16 (4) weeks (range 12–24 weeks) apart, children with CP grew significantly and gained in mass, height and shank length. During clinical examination passive dorsiflexion improved with the knees in flexion [6° (11°), *p* = 0.048] and extension [4° (8°), *p* = 0.076], while significance was only noted with flexed knees.

**Table 1:** Anthropometrics, clinical exam and parameters of gait of typically developing children (TD) and children with cerebral palsy (CP) before and change (post–pre) after bracing

	TD	CP		CP post bracing		
	Mean (SD)	Mean (SD)	ES	Mean Δ	95 % CI	ES
Anthropometrics						
Age (months)	114 (31)	125 (36)	0.2	3.7^††^	[3.2, 4.2]	2.3
Height (cm)	137.7 (15.3)	140.0 (17.8)	0.1	1.5^††^	[1.0, 2.0]	1.6
Shank length (cm)	33.4 (4.5)	33.4 (4.8)	0.0	0.6^†^	[0.0, 1.2]	0.5
Mass (kg)	32.7 (13.0)	38.6 (18.1)	0.4	1.3^††^	[0.6, 2.0]	1.0
PRoM (°)						
Popliteal angle	8 (10)	36 (10)**	2.3	−4	[−1, 10]	0.4
Knee extension	6 (4)	3 (6)	0.6	0	[−2, 2]	0.1
Dorsiflexion (knees flexed)	29 (8)	8 (11)**	2.3	6^†^	[0, 11]	0.5
Dorsiflexion (knees extended)	15 (5)	2 (10)**	1.7	4	[0, 8]	0.5
Plantarflexion (knees extended)	39 (5)	42 (8)	0.4	1	[−1, 4]	0.3
MAS (0–4)						
Plantarflexor tone (knees flexed)	0.0 (0.0)	1.7 (0.9)**	2.7	0.1	[−0.3, 0.5]	0.2
Plantarflexor tone (knees extended)	0.0 (0.0)	2.3 (1.1)**	3.1	0.2	[−0.6, 0.2]	0.2

*PRoM* passive range of motion, *MAS* muscle tone on modified Asworth Scale, *SD* standard deviation, *ES* effect size (Cohen’s d), *CI* confidence interval

* Significant differences between TD and CP with *p* < 0.05 (** p < 0.01)

^†^Significant differences between pre and post bracing in CP with *p* < 0.05 (^††^ *p* < 0.01)

### Morphometrics

**Fig. 3:**
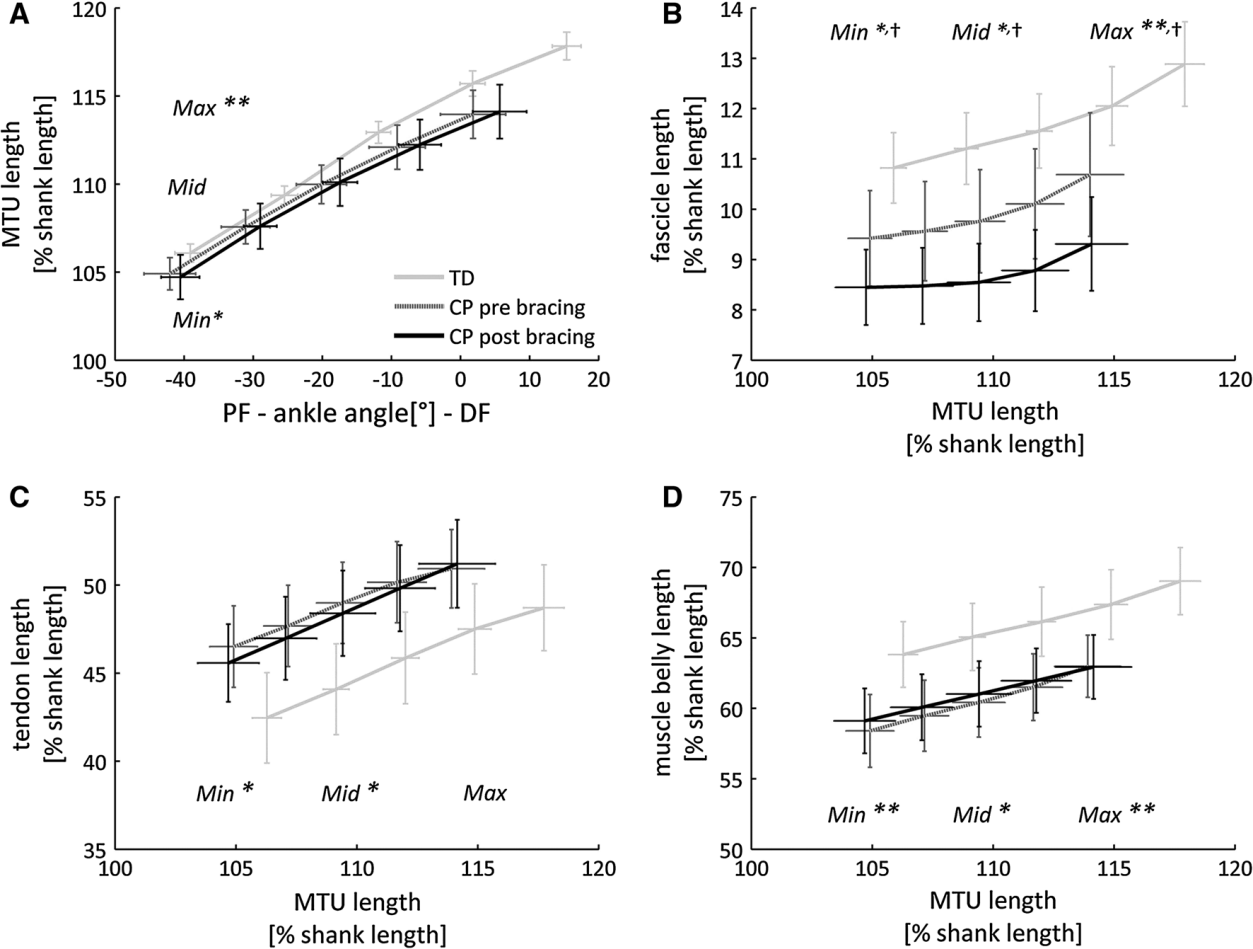
Normalised muscle morphometrics during stretch. Data are group mean (1 SEM). *Significant differences between typically developing children (TD) and children with spastic cerebral palsy (CP) with *p* < 0.05 (**p < 0.01), and ^†^significant differences between pre and post bracing in CP with *p* < 0.05 tested at minimum (Min), matched midrange (Mid) and maximum (Max) muscle–tendon unit length

In children with CP, mean knee flexion angle during scans was 9° (5°) in CP vs. 5° (4°) in TD (*p* = 0.015). Thus, similar *L*_MTU_ were reached at different ankle angles due to altered knee alignments (Fig. 3). The midrange *L*_MTU_ from CP (109.4 % shank length) corresponded to 23° (8°) and 25° (6°) plantarflexion in CP and TD.

**Fig. 4:**
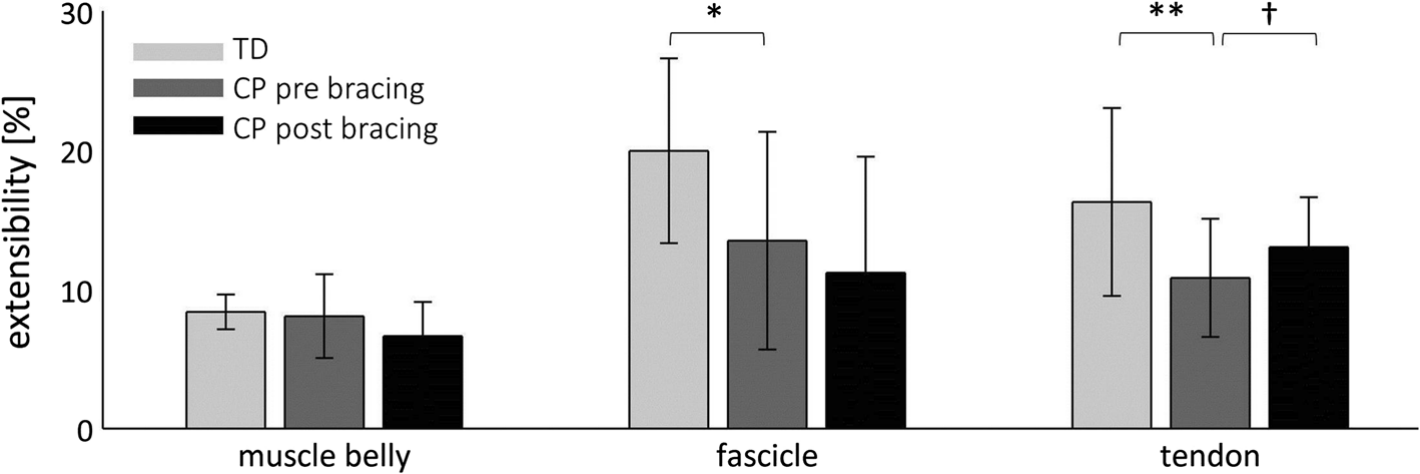
Extensibility of the muscle belly, fascicle and tendon. Data are group mean (1 SD). * Significant differences between typically developing children (TD) and children with spastic cerebral palsy (CP) with *p* < 0.05 (**p < 0.01). ^†^Significant differences between pre and post bracing in CP with *p* < 0.05

Average *L*_MTU_–*L*_FASC_, *L*_MTU_–*L*_TEND_ and *L*_MTU_–*L*_MB_ relationships are plotted in Fig. 3. Detailed statistics can be found in Table 2. Prior to bracing, the total MTU extensibility in CP with respect to controls was reduced by 22 % (*p* = 0.002). As illustrated in Fig. 4, this was accompanied by less fascicle (−32 %) and tendon (−34 %) extensibility (both* p* ≤ 0.014). *L*_MB_ and *L*_FASC_ were significantly shorter throughout the stretch (all *p* ≤ 0.035). *L*_TEND_ was significantly longer at minimum and midrange *L*_MTU_ stretch (both* p* ≤ 0.039). At midrange*, L*_MB_ and *L*_FASC_ were 7 % (*p* = 0.016) and 14 % (*p* = *0.032*) shorter, while *L*_TEND_ was 11 % (*p* = 0.013) longer. MT was thinner, most pronounced (12 %) during the minimum MTU stretch (*p* = 0.027), and FA appeared to be comparable between TD and CP children.

**Table 2:** Normalised muscle morphometrics of typically developing children (TD) and children with spastic cerebral palsy (CP), as well as changes after bracing (post–pre) in CP

	Degree of MTU stretch	TD	CP		CP post bracing		
		Mean (SD)	Mean (SD)	ES	Mean Δ	95 % CI	ES
MTU length [% shank]	Min	106.1 (1.2)	104.9 (2.1)*	0.7	−0.2	[−1.6, 1.2]	0.1
	Matched mid	109.4 (0.0)	109.4 (2.3)	–	–	–	–
	Max	117.8 (1.8)	114.0 (3.1)**	1.6	0.2	[−1.7, 2.0]	0.2
Muscle belly length [% shank]	Min	63.8 (5.2)	58.4 (5.8)**	1.0	0.7	[−0.7, 2.0]	0.3
	Matched mid	65.2 (5.5)	60.4 (5.5)*	0.9	0.5	[−0.7, 1.7]	0.2
	Max	69.1 (5.4)	63.0 (4.9)**	1.2	0.1	[−1.4, −1.4]	0.0
Muscle belly thickness [% shank]	Min	4.4 (0.5)	3.9 (0.8)*	0.8	−0.3	[−0.6, 0.0]	0.5
	Max	4.2 (0.5)	3.7 (0.8)	0.7	−0.3^†^	[−0.6, −0.1]	0.6
Fascicle length [% shank]	Min	10.8 (1.6)	9.4 (2.1)*	0.7	−1.0^†^	[−2.0, −0.1]	0.6
	Matched mid	11.3 (1.6)	9.8 (2.3)*	0.7	−1.1^†^	[−2.2, −0.1]	0.6
	Max	12.9 (1.9)	10.7 (2.7)**	0.8	−1.5^†^	[−2.5, −0.2]	0.6
Fascicle angle [°]	Min	24.3 (4.1)	25.0 (4.9)	0.2	1.0	[−1.5, 3.6]	0.2
	Max	19.2 (3.2)	21.4 (5.7)	0.5	0.7	[−2.0, 3.3]	0.1
Tendon length [% shank]	Min	42.4 (5.7)	46.4 (5.0)*	0.7	−0.9	[−2.5, 0.8]	0.3
	Matched mid	44.2 (5.5)	49.0 (5.2)*	0.9	−0.5	[−1.7, 0.7]	0.2
	Max	49.0 (5.4)	51.3 (5.1)	0.4	0.1	[−1.8, 1.9]	0.0

Matched mid: parameters at midrange MTU length that refers to 50 % MTU stretch in CP

*SD* standard deviation, *ES* effect size (Cohen’s d), *CI* confidence interval

* Significant differences between TD and CP at* p* < 0.05 (** p < 0.01)

^†^Significant differences between pre and post bracing in CP at *p* < 0.05

After bracing, the *L*_MTU_, *L*_MB_ and *L*_TEND_ did not significantly change (all *p* ≥ 0.272), but *L*_FASC_ was significantly shorter throughout the stretch (all *p* ≤ 0.035). At matched midrange *L*_MTU_ stretch, 11 % of *L*_FASC_ was lost with respect to baseline. Simultaneously, MT decreased by 8 %, reaching significance at maximum MTU stretch (*p* = 0.018), while FA showed minor change. No significant changes in fascicle and muscle extensibility were noted (*p* > 0.104), whereas tendon extensibility increased by 20 % (*p* = 0.017).

### 3D gait analysis

Results are shown in Table 3. Before bracing, children with CP walked 16 % slower while taking 13 % shorter steps than TD and landed with a significantly steeper foot contact (all *p* ≤ 0.001). Average constraints in knee extension (−3°) and dorsiflexion (−4°) in stance did not reach significance (*p* ≥ 0.102). Obstructions in dorsiflexion were more pronounced during swing (−5°, *p* = 0.004). Ankle moments in early stance were pathologically increased, whereas ankle moments and power used for propulsion were considerably diminished (all *p* ≤ 0.002). After bracing, walking speed significantly increased by 8 % (*p* = 0.014), while children tended to take longer steps (3 %, *p* = 0.068). Children landed with a significantly better foot-to-floor angle (*p* = 0.006) and the average pattern changed towards heel-toe gait. Dorsiflexion gains in stance failed to reach significance (+2°, *p* = 0.073), but showed significant increases in swing (+2°, *p* = 0.045). The pathologically increased ankle moment during early stance developed towards reference values (*p* < 0.001). While propulsive ankle moments were reduced (*p* = 0.013), ankle power was not significantly changed (*p* = 0.550).

**Table 3:** Results of 3DGA of typically developing (TD) and children with cerebral palsy (CP) before and change (post–pre) after bracing

	TD	CP	ES	CP post bracing		
	Mean (SD)	Mean (SD)		Mean Δ	95 % CI	ES
vel (non. dim.)	0.47 (0.06)	0.40 (0.08)**	1.1	0.03^†^	[0.01,0.06]	0.5
step length (non. dim.)	82.2 (7.3)	71.2 (11.5)**	1.1	2.2	[−0.2, 4.5]	0.4
landing angle (°)––foot to floor	11 (6)	1 (7)**	1.5	2^†^	[1, 4]	0.6
knee flexion (°)––midstance	6 (4)	9 (9)	0.5	1	[−1, 3]	0.1
dorsiflexion (°)––late stance	12 (4)	8 (8)	0.5	2	[0, 4]	0.4
dorsiflexion (°)––swing	6 (3)	1 (6)**	0.9	2^†^	[0, 4]	0.4
ankle moment (Nm/kg)––early stance	0.6 (0.1)	0.9 (0.2)**	1.3	−0.1^††^	[−0.2, −0.1]	0.8
ankle moment (Nm/kg)––late stance	1.3 (0.2)	1.1 (0.2)**	1.0	−0.1^†^	[−0.2, 0.0]	0.5
ankle power (W/kg)––late stance	3.9 (0.9)	2.0 (0.7)**	2.4	0.1	[−0.1, 0.2]	0.1

*3DGA* instrumented 3D gait analysis, *SD* standard deviation, *ES* effect size (Cohen’s d), *CI* confidence interval

* Significant differences between TD and CP with *p* < 0.05 (** p < 0.01)

^†^Significant differences between pre and post bracing in CP with *p* < 0.05 (^††^ *p* < 0.01)

## Discussion

This study set out to provide information about gastrocnemius muscle morphometrics in children with CP before and after a period of ankle foot bracing while referencing to untreated typically developing (TD) peers. Our assumption was that the spastic medial gastrocnemius (MG) would change towards TD and lengthen after a period of ankle–foot bracing. However, no significant gains of muscle belly (*L*_MB_) or tendon (*L*_TEND_) length occurred and fascicle length (*L*_FASC_) further shortened while muscle bulk decreased. Nonetheless, on a joint level, significant increases in passive dorsiflexion were noted. This primarily affected dorsiflexion with knees flexed, but the majority of the children also gained dorsiflexion when assessed with extended knees. During gait, children walked faster, and in particular, dorsiflexion during swing, as well as the foot landing pattern improved.

### Muscle morphometrics prior to bracing

Before bracing, shortening of the spastic MG *L*_MB_ and *L*_FASC_ was pronounced in CP and maximum values during stretch only approached minimum values of TD. This displays considerable atrophy among relatively high functioning, independently ambulant children and youth (GMFCS I and II) with CP. As described before, muscle thickness (MT) was less [9] and fascicle angle (FA) appeared to be similar [13, 14]. Shorter *L*_FASC_ and *L*_MB_ also agree with recent investigations [11, 12]. Besides, our data confirm that MG fascicles are less extensible than usual [32]. Therefore, it appears reasonable to assume that spastic MG fibres may lack sarcomeres in series or that they might contain longer, already drawn-out sarcomeres, as had been shown for forearm or hamstring muscle [16, 17]. Potentially, more connective tissue could also impede the actual extensibility of the fibres [17]. Whether these alterations are caused by changed muscle growth or result as a consequence of decreased loading and reduced physical activity remains a subject of controversy [33]. In contrast to *L*_MB_ and *L*_FASC_, at midrange stretch, *L*_TEND_ was longer than usual [10]. Even though *L*_TEND_ at similar degrees of muscle–tendon unit (MTU) stretch was longer, its total extensibility seemed to be compromised. In summary, if clinicians or therapists want to improve MG muscle pathology in children with CP, growth of muscle belly in length and thickness, as well as longer *L*_FASC_, appear to be desirable goals. Longer *L*_FASC_ with more sarcomeres in series could in principle promote function by increasing the muscle’s contractile velocity and enable a muscle to exert force over a larger joint RoM [34].

### Muscle morphometrics after bracing

Conversely, after bracing, an additional 11 % in *L*_FASC_ was lost at matched amounts of MTU stretch and MT decreased by 8 %. This is the first study to provide information about spastic calf morphometrics after brace wear. Previous investigations on the longitudinal change of calf muscle morphometrics were also done on invasive treatments with worse outcome: despite improving passive dorsiflexion, surgical gastrocnemius recessions induced shortening of *L*_MB_ [35] and 32 % shortening of *L*_FASC_ [36]. Botulinum toxin injections caused reductions in MT of ~12 % [37]. While we observed shorter *L*_FASC_, the *L*_MB_ modifications seemed negligible. Due to its pinnated fibre arrangement, loss in *L*_FASC_ may not be reflected by loss in *L*_MB_, if, as observed, the MT reduces too. Although we did not instrumentally assess the force-producing capability of the MG, these architectural deteriorations suggest that the muscle would have gotten weaker.

### Potential causes for morphometric changes

A potential cause for shorter *L*_FASC_, and thus progressive muscle contracture, could be that muscle tissue indeed failed to keep up with bone growth [2]. For normalization, *L*_FASC_ was set in relation to the shank length [11]. In TD children, MG *L*_FASC_ usually grows in proportion with the tibia [38]. During the current intervention, the shank of children with CP grew by 1.8 %, while unscaled *L*_FASC_ dropped by 0.3 cm. Hence, these atrophic changes do not solely expose a lack of scaling to bone growth.

Another reason for shorter *L*_FASC_ could be adaptations of the tendon. Although no significant changes in fascicle and muscle extensibility were noted, the extensibility of the tendon increased. Still, we did not observe changes in *L*_TEND_ defined as a straight line from the heel marker to the MTJ. However, in absence of instrumented measures for the applied tension during passive stretch, this measure ignores slack. Consequently, we generally underestimated *L*_TEND_ at small degrees of MTU stretch and overestimated its extensibility. Slack is usually surpassed shortly beyond neutral ankle alignment in TD [32]. In our data, only 2–3 % of tendon extensibility in TD children would be noted above that point, which confirms previous reports about intrinsic tendon tissue strain [39]. Moreover, during maximum MTU stretch, when all slack is taken up, no changes in *L*_TEND_ of children with CP were noted, which could suggest that no major changes in *L*_TEND_ occurred. Assuming that the tendon could have initially gotten more compliant during bracing, such as observed in growing animals [23, 24], the spastic fascicles were unstrained, which can trigger loss of sarcomeres [21, 22] and would fit the reductions in *L*_FASC_. Although intrinsic tendon properties are cumbersome to measure, more detailed information is necessary to clarify this.

Worth mentioning, the MG tendon also integrates the run-out from the deep MG aponeurosis and the achilles tendon to which both gastrocnemius and soleus merge. With the soleus fascicles also attaching distal to the MG’s muscle–tendon junction (MTJ), the increase in MG tendon extensibility could reflect a more compliant soleus as well. This also explains the significant increase of passive dorsiflexion with the knee held in flexion. Most ultrasound research in CP is currently done on gastrocnemius morphometrics, probably due to its superficial position. Clearly, more information about the soleus architecture in equinus needs be gathered.

The current outcome could also be attributed to the bracing regime. Overall, the MG is highly susceptible to disuse atrophy [40]. Since ankle motion in the brace was also largely restricted, a possible reason for the loss in *L*_FASC_ and the decreased MT could be decreased muscle ecxcursion, which is important in regulating sarcomere number in growing animals [41]. Besides, as all children likely slept with bent knees, the ankle–foot brace may have not provided sufficient stretch on the bi-articular gastrocnemius. Surely, a knee-ankle–foot brace would be logic to target gastrocnemius contracture. Sees and Miller [42] recently emphasised this, elsewise suspecting contracture of the gastrocnemius to worsen. Our results reinforce this. As a final point, considering that prior to bracing, average dorsiflexion with knees extended in the current study group was 2°, use of augmented force into further dorsiflexion seemed critical. Such an extensive bracing treatment is very demanding and may cause insufficient compliance. That may be one reason why below-knee casting or nighttime ankle–foot orthotics are often being used [7, 8].

### Functional benefits of bracing

Next to improving passive dorsiflexion with flexed knees, this ‘positional’ bracing also prevented deterioration in passive dorsiflexion with the knee in extension in 76 % of the children (13 of 17). The average dorsiflexion gain of 4° with extended knees marginally failed to reach significance. In the past, below-knee serial casts have also been shown to only increase passive dorsiflexion with flexed knees [8]. Based on the progressive loss of passive dorsiflexion during CP childhood [6], these results appear to be a beneficial outcome for children with CP! More importantly, from a functional perspective, the children walked faster and their ankle kinematics improved primarily in swing. Positioning the foot better for landing can be vital to avoid tripping and to prevent mid-foot break deformities. Reduced ankle moments during early stance may display less pathological dynamic joint stiffness after bracing. However, the reduced moments for push-off may be a side effect, but are in accordance with reduced muscle thickness.

We think that these functional gains outweigh the atrophic effects on muscle morphometrics. Restoring dorsiflexion and normalizing muscle morphometrics may not necessarily occur in concert. By concurrently improving morphometrics, a larger or potentially more sustainable change in function may be achieved. Coordinative and neural aspects may of course also modulate the direct relations between morphometrics and function. These aspects should be content of future interventions. Most likely this would include activities such as calf strength training [43] or instrumented cyclic stretching [20].

### Considerations for ultrasound scans

Ultrasound scans are frequently used to study muscle architecture in CP. To perform valid comparisons between TD and CP, morphometrics should be assessed at similar muscle states. Some studies extracted MG parameters at resting or neutral ankle position without detailed info on knee alignment or at PRoM limits only [13, 14, 37]. Recently, a common ankle angle was suggested [12]. Still, even at similar ankle alignment, our data shows that the MTU can be considerably shorter in CP. Albeit referencing to *L*_MTU_, we found no common overlap. Possibly, we could have done so when allowing for semi-flexed knees [12], but the MG would then be slightly off-tension. By using a calculation of the entire MTU path, we accounted for different knee angles during testing, but the issue of standardization may depend on patient positioning. MG scans are mostly done when lying prone [10, 11, 14, 20, 38], but also when lying supine [19] or when sitting [12]. The latter probably poses most difficulties to achieve straight knees in case of short hamstrings. With the current setup, it seemed best choice to compare TD at the average midrange *L*_MTU_ of the children with CP.

### Limitations

First, we investigated a convenience sample and can only speculate about untreated natural progression of contracture. The treatment duration was somewhat variable depending on the childrens’ outpatient attendance, and not every child received full-time bracing. Longer treatment duration explained 15 % of the loss in *L*_FASC_ assessed by simple linear regression (*R*^2^). Besides, effects on *L*_FASC_ were not different between the bracing protocols (*p* = 0.580). In the future, the separate impact of daytime and nighttime orthotics should be quantified. Additionally, the applied tension during stretch was not instrumentally standardised. Nevertheless, it seems very unlikely that shorter *L*_FASC_ after bracing could be attributed to consistently reduced manually applied tension, since *L*_FASC_ was shorter during the entire MTU stretch.

## Conclusions

To the best of our knowledge, this is the first study about calf morphometrics in CP after a non-invasive orthotic treatment. Prior to bracing, the children with CP had shorter and thinner MG muscle bellies, and shorter fascicles but longer distal tendons than controls. Positional ankle–foot braces significantly improved passive dorsiflexion with the knees flexed and improved the gait pattern of the children, but failed to improve MG morphometrics. Further shortened fascicles and thinner muscle bellies are likely due to the fact that bracing potentially decreased the MG excursion or kept the bi-articular muscle off-tension. Theoretically, braces may need to extend the knee if MG morphometric pathology is to be targeted. Promoting dorsiflexion and normalizing muscle morphometrics seems difficult to be accomplished in concert using traditional orthopaedic means such as surgery, botulinum toxin injections or brace wear in isolation. Consequently, there is a need for concomitant treatments that promote muscle growth.

## Abbreviations

CP: Cerebral palsy
MG: Medial gastrocnemius
TD: Typically developing peers
MTU: Muscle-tendon unit
MAS: Modified Ashworth scale
MTJ: Muscle-tendon junction
FA: Fascicle angle
*L*_MTU_: Muscle-tendon unit length
*L*_FASC_: Fascicle length
*L*_MB_: Muscle belly length
*L*_TEND_: Tendon length
GMFCS: Gross motor function classification system
PRoM: Passive range of motion
3DGA: 3D gait analysis
